# Cognitive development in patients with Mucopolysaccharidosis type III (Sanfilippo syndrome)

**DOI:** 10.1186/1750-1172-6-43

**Published:** 2011-06-20

**Authors:** Marlies J Valstar, Jan Pieter Marchal, Martha Grootenhuis, Vivian Colland, Frits A Wijburg

**Affiliations:** 1 Department of Pediatrics and Amsterdam Lysosome Center 'Sphinx', Academic Medical Center, University of Amsterdam, Amsterdam, The Netherlands; 2 Psychosocial Department, Emma Children's Hospital, Academic Medical Center; University of Amsterdam, Amsterdam, The Netherlands

## Abstract

**Background:**

Mucopolysaccharidosis type III (MPS III, Sanfilippo syndrome) is a lysosomal storage disorder caused by a deficiency of one of the enzymes involved in the degradation of heparan sulfate.

MPS III is characterized by progressive mental deterioration resulting in severe dementia. A number of potentially disease-modifying therapies are studied. As preservation of cognitive function is the ultimate goal of treatment, assessment of cognitive development will be essential in order to evaluate treatment efficacy. However, no large scale studies on cognitive levels in MPS III patients, using formal psychometric tests, have been reported.

**Methods:**

We aimed to assess cognitive development in all 73 living patients with MPS III in the Netherlands.

**Results:**

Cognitive development could be assessed in 69 patients. In 39 of them developmental level was estimated > 3 months and formal psychometric testing was attempted. A remarkable variation in the intellectual disability was detected.

**Conclusions:**

Despite special challenges encountered, testing failed in only three patients. The observed broad variation in intellectual disability, should be taken into account when designing therapeutic trials.

## Background

Mucopolysaccharidosis type III (MPS III, Sanfilippo syndrome) is an autosomal recessive lysosomal storage disorder caused by a deficiency of one of the four enzymes involved in the degradation of the glycosaminoglycan heparan sulfate. Four subtypes of MPS III, caused by deficiency of one of these four enzymes, are recognised.: MPS III types A, B, C and D. Signs and symptoms and course of the disease in the different subtypes are, however, indistinguishable.

MPS III is a neurodegenerative disorder characterized by an initial symptom free period followed by progressive intellectual decline, finally resulting in severe dementia. Severe behavioural problems are a predominant symptom in most patients, characterized mainly by extremely hyperactive behaviour [1]. Other symptoms include sleeping problems, recurrent diarrhoea, frequent ear, nose and throat infections, hearing and visual impairment and epilepsy. Patients usually die at the end of the second or the beginning of the third decade of life, although longer survival has been reported in patients with an attenuated form of MPS III[2-4].

The rate of decline of intellectual capacities varies widely in patients with MPS III [2,5-8]. Patients with the severe or 'classical' phenotype of MPS III have a normal to near normal development during the first two years of life, followed by a slowing of development and full stagnation of development at around the age of 3 to 4 years, and finally regression of cognitive capacities [6,9]. Patients usually become fully dependent on care early in their teenage years.

Patients with a more attenuated phenotype may have a stable intellectual disability for many years. These patients, who also have a longer preservation of motor functions, may live well into adulthood [8,10,11].

Testing the level of cognitive development in patients with MPS III is necessary in order to quantify the decline of intellectual abilities. However, no studies using formal psychometric testing in a large group of unselected patients with MPS III have been reported yet. This information on the natural course is important as new disease-modifying therapies for MPS III are currently developed with preservation of cognitive function as the main therapeutic goal[12-14].

We therefore aimed to assess the cognitive development in all living MPS III A, B and C patients in the Netherlands, and provide data from this relatively large and unbiased cohort.

## Methods

### Recruitment of patients

All living patients ever diagnosed with MPS III in the Netherlands since enzyme testing became available (1967) were identified by retrieving the data from the combined registry of the four diagnostic centres in the Netherlands [15]. Parents or legal representatives were asked to participate in this study via physicians who had requested the initial diagnostic studies. The study was approved by the Medical Ethical Committee of the AMC, Amsterdam.

### Developmental assessment

Developmental assessment was done by a single psychologist (JPM), experienced in testing patients with intellectual disabilities. Patients were tested using an appropriate test for their estimated developmental age. All patients with an estimated developmental age above 3 months were tested within the scope of this study.

To exclude any effects of training, testing was performed at least one year after a previous developmental test, if applicable. To optimize testing in these patients with often behavioral problems in addition to the intellectual disabilities, patients were tested in a familiar environment (at home or at their day care centre).

Three different developmental tests were used. All tests were performed according to their guidelines.

The Dutch version of the Bayley Scales of Infant Development II (BSID-II-NL) was used in patients with an estimated developmental age between 4 months and 3 years [16]. The Snijders-Oomen Nonverbal intelligence test - Revised (SON-R 2 1/2-7), a validated Dutch non-verbal developmental test, was used in patients with an estimated developmental age between 3 years and 6 years [17]. Finally, the Dutch version of the Wechsler Intelligence Scale for Children, third edition (WISC-III-NL) was used in patients with an estimated developmental age above 6 years [18].

The BSID-II-NL was chosen as it is the most recent version of the Bayley Scales of Infant Development available and validated in the Netherlands. It is considered a reliable and valid test by COTAN, the Test Advisory Board for the Dutch Psychologists Institute, and is frequently used in research. The Snijders-Oomen Nonverbal Intelligence Test - Revision (SON-R 2.5-7) was chosen as it received a good evaluation from COTAN and is available and validated in Dutch. The internationally often used Wechsler Preschool and Primary Scale of Intelligence, an alternative for SON-R, was considered unreliable in Dutch by COTAN and at the time of this study the Dutch version of the Third Edition of the was not yet released. Finally, the WISC-III-NL was used as this is the most recent version of the Wechsler Intelligence Scale for Children available in the Netherlands. The WISC-III-NL is considered reliable and valid by COTAN.

All tests have been used previously in studies on cognitive development in children and adolescents with intellectual disabilities [19].

## Results

### Patients

In total, 73 MPS III patients were alive at the time of this study.

**Figure 1:**
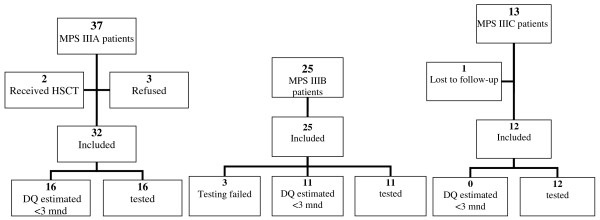
**Flow diagrams of MPS III patients included in the study**.

Thirty-seven patients had MPS IIIA. Of them, 32 were assessed for inclusion in this study as two patients who had received a Hematopoietic Stem Cell Transplantation (HSCT) were excluded and families of three other patients refused to participate. Twenty-three patients had MPS IIIB. All patients participated in the study. Thirteen MPS IIIC patients were alive at the time of the study. One MPS IIIC patient was lost to follow-up, while the remaining 12 patients participated in this study. In total, 69 patients were assessed for feasibility for developmental testing (Figure 1).

All patients were untreated, except for three patients (2 patients with MPS IIIA for less than 6 months and one patient with MPS IIIC for a period of 3 years), taking genistein, a soy-bean derived isoflavone, which may ameliorate disease severity in MPS III by inhibition of GAG synthesis [20].

### MPS IIIA patients

**Figure 2:**
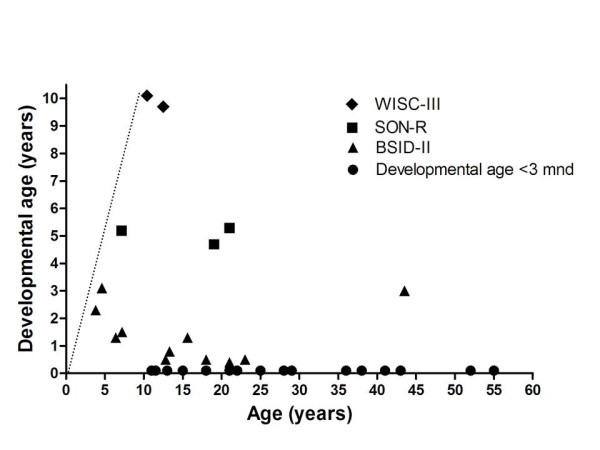
**Developmental age in patients with MPS IIIA (n = 33)**. The straight dotted line indicates the normal developmental pattern.

Of the participating 32 MPS IIIA patients, 16 patients were assessed as having a developmental age below 3 months and were excluded for further testing. In the remaining 16 patients, the WISC-III-NL was performed in 2 patients, the SON-R 2 1/2-7 in 3 and BSID-II-NL in 11. Results are shown in Figure 2.

### MPS IIIB patients

**Figure 3:**
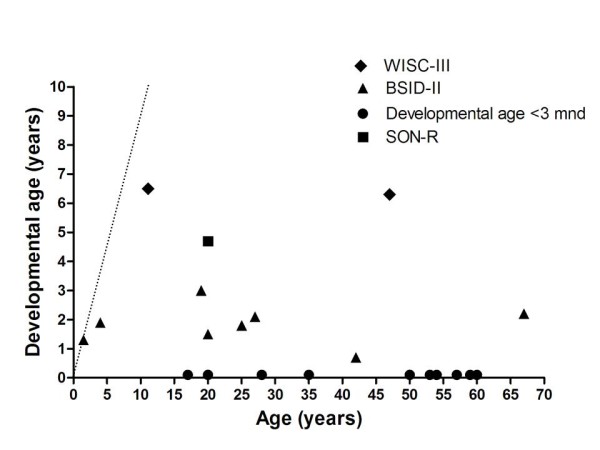
**Developmental age in patients with MPS IIIB (n = 23)**. The straight dotted line indicates the normal developmental pattern.

Eleven patients of the 25 participating MPS IIB patients were assessed as having a developmental age below 3 months. Testing was attempted in all remaining 12 patients. Testing failed in three of them due to extreme anxiety, aggressive behavior and refusal to cooperate. These patients were excluded. Of the remaining 9 patients, the BSID-II-NL was performed in 8 patients and the SON-R 2 1/2 - 7 in 1 patient and WISC-III-NL in 2 patients. Results are shown in Figure 3.

### MPS IIIC patients

**Figure 4:**
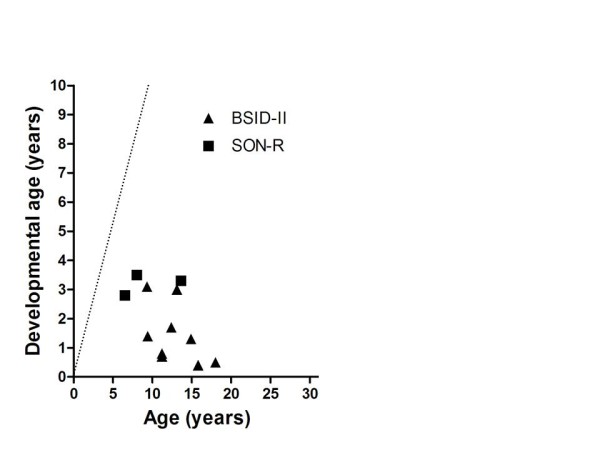
**Developmental age in patients with MPS IIIC (n = 12)**. The straight dotted line indicates the normal developmental pattern.

All 13 participating MPS IIIC patients had a developmental age above 3 months. BSID-II-NL was performed in 9 patients and the SON-R 2 1/2-7 in 3 patients. Results are shown in Figure 4.

### Difficulties in cognitive testing in patients with MPS III

Patients in our study frequently showed extreme hyperactive behavior and fidgeting. This often resulted in noisy behavior, throwing of test material, mouthing and biting of test materials and unwillingness to perform specific tasks.

Hyperactive behavior in MPS III patients is complicated by additional attention deficits. Patients are easily distracted and can frequently only perform short tasks, further complicated by motor apraxia, which was observed in a number of patients. This was particularly problematic in specific assignments necessitating more detailed instructions. Patients were found to be much more interested in persons compared to the materials used in the tests, further complicating testing.

Aggressive behavior may be an additional symptom in MPS III patients. In this cohort, severe aggressive behavior was only encountered in one patient with MPS IIIB and resulted in failure to test.

Excessive anxiety is also a symptom in MPS III patients. In this study, anxiety resulted in failed testing in one patient with MPS IIIB.

Stereotypic behavior and stereotypic language were seen in several patients. Stereotypic language in combination with aphasia significantly complicated language assessment in these patients. In addition, many patients tended to perseverate.

Developmental testing was also hampered by physical disabilities in several patients. One patient suffered from retinitis pigmentosa resulting in a decreased vision, complicating testing.

Finally, a number of patients showed a disharmonic developmental pattern. This could be reliably assessed in all patients tested with the WISC-III-NL and SON-R 2 1/2-7 and for patients tested with the BSID-II-NL test with a developmental age between 12 and 30 months. In 23% of patients the disharmonic developmental pattern was found to be significant, according to the test guidelines.

### Discussion and conclusions

**Figure 5:**
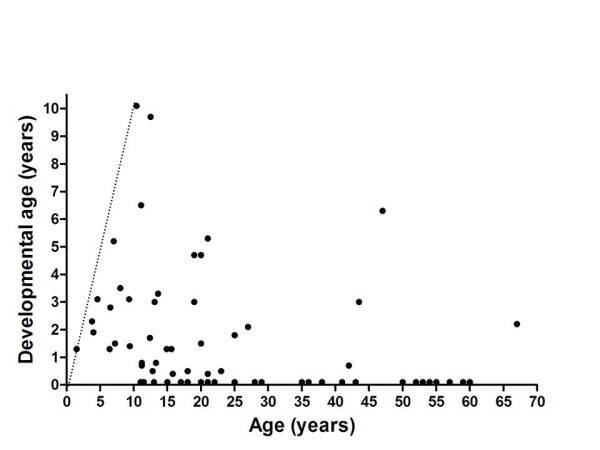
**Developmental age in all tested patients with MPS III according to age**. The straight dotted line indicates the normal developmental pattern.

We assessed cognitive development in 69 patients with MPS III and we could perform formal assessment of psychometric developmental in 39 patients with an estimated developmental level > 3 months. A remarkable variation in the intellectual disability was detected. Patients with the classical severe phenotype of MPS III generally reached a maximal developmental age of approximately 3-4 years (Figure 5), while patients with a more attenuated phenotype showed a considerably wider spectrum with some patients, reaching even a developmental age of 10 yrs in one patient. Patients with an attenuated phenotype, especially MPS IIIB patients, may have a long period of stable intellectual disability even well into adulthood, without any signs of regression [4].

Formal assessment of cognitive functions in patients with MPS III poses several major challenges. Hyperactive behavior, anxiety and aggressive behavior are a predominant symptom in MPS III [1,21]. Concurrent concentration problems [1], present in the majority of patients with MPS III, further complicate testing and assignments requiring longer instructions proved to be difficult for most patients. A test such as the BSID-II, which consists of many separate short tasks, was found to be the most suitable for these easily distracted patients. The more recently developed BSID-III appears to be even more suitable for testing this group of patients **a**s it consists of five distinct scales of development (Cognitive, Language, Motor, Social Emotional and Adaptive Behavior Scale), while the BSID-II consists of three scales (Mental, Motor and Behavior Scales). However, this test was not validated in Dutch at the time of this study.

Twenty-three percent of the tested patients showed a significant disharmonic developmental pattern. This phenomenon is often detected in patients with organic mental retardation. For example in patients with trisomy 21 speech is usually spared, even in the presence of extensive dementia [22]. A disharmonic profile is characteristic in patients with MPS III, as intellectual decline almost invariably precedes regression of motor functions [2,6,23].

Developmental testing may be hampered by physical disabilities observed in MPS patients [21]. In our cohort this was not a significant problem. Some patients showed mild hearing loss, but this did not complicate testing. Only one patient, with visual disabilities due to retinitis pigmentosa, could not complete some of the test assignments due to a physical disability.

Despite the challenges encountered in assessing cognitive developmental in MPS III patients, developmental testing failed in only three. Failure was due to extreme anxiety, severe aggressive behavior and refusal to cooperate. In all other patients, parents and care-givers expressed the opinion that patients showed a reliable reproduction of their development during the assessment.

Testing in a familiar environment such as home or day-care center is likely to improve the success of psychometric testing in patients with MPS III. Patients with MPS III are easily distracted and any change in environment can increase behavioural disturbances, resulting in an autistic behavior [24].

Developmental assessment in untreated patients with MPS III characterizes the natural history of MPS III [21], which appears to be remarkably variable. Patients with the severe MPS III phenotype show a slowing of development from the age of 2-4 years onwards. Subsequently, a maximal developmental level of approximately 3.5-4 years is reached followed by a regression of developmental level from the age of 4-6 years [6,9]. Although this phenotype is the most well known and reported phenotype of MPS III, an increasing number of patients with an attenuated course of MPS III is reported [3,4,7,8,10,11].

A limitation of our study is that we only obtained cross-sectional data on cognitive development in MPS III. Longitudinal studies in patients with different MPS III subtypes and different phenotypic severity will be needed to be able to assess efficacy of treatment in individual patients. In addition, we did not study hyperactive and agressive behavior and anxiety, which are common behavioral symptoms in MPS III as our study was focused only on cognitive development. Behavioral disturbances may also influence the reliability of cognitive testing. By testing the patients in a familiar environment which often reduces the behavioral problems, we tried to reduce this potential confounder as much as possible.

Currently, several disease-modifying treatments for MPSIII are studied. These therapies include substrate reduction therapy, HSCT, intrathecal enzyme replacement therapy and gene therapy [25]. In order to achieve optimal efficacy, treatment should be initiated before irreversible CNS damage occurs. Early diagnosis is therefore essential. Increasing awareness of MPS III in the medical community may help to diagnose MPS III patients as early as possible. However since developmental delay is usually the presenting symptom in MPS III patients, CNS damage is almost invariably present at diagnosis. Therefore, identification of patients in the pre-symptomatic period can only be achieved by screening of patients at risk (e.g. sibs) or by newborn screening. A major criterion for inclusion of a disease in screening programs is adequate knowledge on the natural history of the disorder [26]. Our study provides information on the spectrum of cognitive disabilities in patients with MPS III, which helps to understand the variability in the natural course of this devastating inborn error of metabolism.

## Competing interests

The authors declare that they have no competing interests.

## Authors' contributions

MJV participated in the study-design, carried out the inclusion of the patients and drafted the manuscript. JPM carried out the developmental testing. MG and VC participated in the study design and coordinated and helped with the developmental testing. FAW designed the study, and participated in the coordination and helped to draft the manuscript. All authors read and approved the final version of the manuscript.
